# Inhibition of Polyglutamine Misfolding with D-Enantiomeric Peptides Identified by Mirror Image Phage Display Selection

**DOI:** 10.3390/biom12020157

**Published:** 2022-01-18

**Authors:** Pauline Elisabeth Kolkwitz, Jeannine Mohrlüder, Dieter Willbold

**Affiliations:** 1 Institute of Biological Information Processing (IBI-7), Forschungszentrum Jülich, 52425 Jülich, Germany; p.kolkwitz@fz-juelich.de (P.E.K.); j.mohrlueder@fz-juelich.de (J.M.); 2 Institut für Physikalische Biologie, Heinrich-Heine-Universität Düsseldorf, 40225 Düsseldorf, Germany

**Keywords:** polyglutamine diseases, protein misfolding diseases, phage display, all-D-peptide therapeutics, protein aggregation

## Abstract

Nine heritable diseases are known that are caused by unphysiologically elongated polyglutamine tracts in human proteins leading to misfolding, aggregation and neurodegeneration. Current therapeutic strategies include efforts to inhibit the expression of the respective gene coding for the polyglutamine-containing proteins. There are, however, concerns that this may interfere with the physiological function of the respective protein. We aim to stabilize the protein’s native conformation by D-enantiomeric peptide ligands to prevent misfolding and aggregation, shift the equilibrium between aggregates and monomers towards monomers and dissolve already existing aggregates into non-toxic and functional monomers. Here, we performed a mirror image phage display selection on the polyglutamine containing a fragment of the androgen receptor. An elongated polyglutamine tract in the androgen receptor causes spinal and bulbar muscular atrophy (SBMA). The selected D-enantiomeric peptides were tested for their ability to inhibit polyglutamine-induced androgen receptor aggregation. We identified D-enantiomeric peptide QF2D-2 (sqsqwstpqGkwshwprrr) as the most promising candidate. It binds to an androgen receptor fragment with 46 consecutive glutamine residues and decelerates its aggregation, even in seeded experiments. Therefore, QF2D-2 may be a promising drug candidate for SBMA treatment or even for all nine heritable polyglutamine diseases, since its aggregation-inhibiting property was shown also for a more general polyglutamine target.

## 1. Introduction

The folding and aggregation of proteins are usually considered to be competing mechanisms [1]. Aggregating proteins are often intrinsically disordered proteins (IDP) [2]. During the aggregation process, monomers of the aggregating protein form small, soluble oligomers which can accumulate into larger aggregates [3]. The most common aggregate structure is the cross-beta amyloid fibril, which has been described at atomic resolution for several examples [4,5,6,7].

Aggregates formed by misfolded proteins can lead to cytotoxic effects [8]. Several neurodegenerative diseases are caused by misfolded proteins, such as Alzheimer’s disease (AD), Parkinson’s disease (PD), prion encephalopathies, amyotrophic lateral sclerosis (ALS) or the heritable polyglutamine diseases, belonging to the triplet repeat diseases [9,10,11]. Several proteins contain sections with a number of consecutive glutamines, referred to as “polyglutamine proteins” or “polyQ proteins” [12,13]. Polyglutamine proteins form amyloids in a nucleated growth mechanism, with the critical nucleus’s size depending on the length of the polyQ chain [14,15,16]. As observed for other amyloid proteins, the fibrils formed by polyglutamine proteins are able to seed, even between different polyQ lengths [17,18].

Today, nine heritable polyglutamine misfolding diseases are known, namely Huntington’s chorea, spinal and bulbar muscular atrophy (SBMA) or Kennedy’s disease, spinocerebellar muscular atrophy 1 (SCA1), SCA2, SCA3 or Machado-Joseph disease, SCA6, SCA7, SCA12, SCA17 and dentatorubral-pallidoluysian atrophy (DRPLA) or Haw River syndrome [19,20,21,22,23,24,25,26,27,28,29]. Still, no effective therapies are known [19]. Analogous to other protein misfolding diseases, the oligomers are thought to be the main toxic species in polyglutamine misfolding diseases [30,31,32].

Given that the misfolding and aggregation of proteins with an elongated polyglutamine tract is the cause of polyglutamine diseases, inhibiting the aggregation process by stabilizing the native conformation of the polyglutamine protein with a therapeutic agent that binds to natively folded polyglutamine-tracts may be a promising therapeutic approach. Peptides as monomer-stabilizing agents may be suitable as polyglutamine binding partners [33], since they offer a much more effective and specific binding to polyglutamine tracts than small molecules. In one example, a polyglutamine-binding peptide was able to suppress neurodegeneration in *Drosophila* [34]. Due to the degradation of peptides by metabolic processes [35], it is advantageous to deliver peptides that exclusively consist of amino acid residues in D-enantiomeric conformation (D-peptides). These all-D-peptides exhibit less immunogenity, if at all, and an elevated proteolytic stability compared to their L-enantiomeric counterparts [35,36].

A mirror image phage display selection can be carried out using a D-enantiomeric target. It follows that the D-enantiomeric “mirror image” of the selected peptide ligand can be synthesized and binds to the native L-enantiomeric target [35,37,38]. It was already shown that this is a successful approach for neurodegenerative diseases. As an example for an all-D-peptide that progressed into clinical development, the D-enantiomeric peptide RD2 improved the cognition and learning ability in three different mouse models for Alzheimer’s disease even by oral administration. It is an example for D-enantiomeric peptides that pass the blood–brain barrier after peripheral administration [39].

In the present study, we aimed to identify a D-enantiomeric peptide ligand of polyglutamine proteins that inhibits their aggregation. To this end, a phage display selection was performed with the mirror image of an androgen receptor fragment containing Q23-polyglutamine. A subset of the phage display selected peptides have been synthesized as D-enantiomeric peptides and were subsequently tested on the fragment of the L-enantiomeric construct of the androgen receptor with an elongated polyglutamine tract or a universal polyglutamine stretch, with respect to their ability to bind and inhibit their aggregation.

## 2. Materials and Methods

### 2.1. Polyglutamine Proteins and Compounds

The synthetic mirror image of the androgen receptor ARQ23^51–96^ was obtained from JPT (JPT Peptide Technologies GmbH, Berlin, Germany) with an N-terminal biotin, connected to the peptide via Trioxatridecan-succinamic acid (ttds)-linker.

The selected all-D-peptide compounds were obtained from Caslo (CASLO ApS, Lyngby, Denmark), whereas L-ARQ46^51–96^ and the universal polyglutamine peptide L-K2Q46K2 were obtained from peptides & elephants GmbH (Henningsdorf, Germany). All peptides used in this study are summarized in Table 1.

### 2.2. Disaggregation of Polyglutamine Proteins

Following the disaggregation protocol that Chen et al. published [40], the polyglutamine proteins were incubated for three days in 1:1 TFA/Hexafluoroisopropanol (HFIP) or overnight in 100% trifluoroacetic acid (TFA). Afterwards the solvent was evaporated with N_2_ gas treatment and the polyglutamine protein redissolved in the experiment buffer.

### 2.3. Mirror Image Phage Display

The mirror image phage display was performed with D-Biotin-ttds-ARQ23 as target that was disaggregated in 100% TFA. The solvent was evaporated by N_2_ gas treatment. The target was redissolved in Tris-buffered saline (TBS) pH 7.5. Furthermore, D-Biotin-ttds-ARQ23 was immobilized on a high capacity streptavidin coated polystyrene plate with “SuperBlock“ by Thermo scientific (Thermo Fisher scientific, Waltham, MA, USA) for 30 min. TBS pH 7.5 with 0.1% Tween20 served as the selection and immobilization buffer. A total of 4 pmol of the target was immobilized on the surface for each selection round. In parallel, Biotin-ttds was immobilized in another well and served as negative control in the empty selection that was performed in parallel to the target selection. The immobilization was followed by blocking with BSA or milk powder (10 mg/mL) and quenching with biotin for 30 min. Alternating blocking agents were used to avoid selection of BSA or milk powder binders, starting with BSA in the first round. In each selection round 6 × 10^11^ Phages were used as input, starting with the Trico 16 library from Creative Biolabs (Lot: CBLX021820; Creative Biolabs Inc., Shirley, NY, USA). The phages were incubated with the target for 30 min. During the three selection rounds, the number of washing steps was increased from six in selection round 1 to 12 washing steps in the third selection round with TBS pH 7.5 with 0.1% Tween20 and 2 mg/mL BSA as washing buffer. The phages were eluted with 100 µL 0.2 M Glycin-HCl (pH 2.2) that was incubated in the well for 10 min at room temperature (RT). Consecutively the solution containing the eluted phages was removed from the plate and mixed with 25 µL 1 M tris-HCl pH 9.1. Of this neutralized phage containing solution (output), 110 µL were added to a 20 mL *Escherichia coli* K12 ER2738 culture that were grown in lysogeny broth (LB)-medium with 20 µL Tetracyclin to an OD_600_ of 0.1. 5 µL of the output were mixed with 95 µL LB medium and a dilution series from 10^−2^ to 10^−8^ was prepared with LB medium. 100 µL *E. coli* K12 E2738 were added to each well and the resulting 200 µL were plated with 800 µL of top agar (10 g Bacto-Trypton, 5 g yeast extract, 5 g NaCl, 1 g MgCl, 7 g agarose, 1 l H_2_O) on 35 × 10 mm plates (Sarstedt, Nümbrecht, Germany) with IPTG-xGal agar (1 µL/mL stock solution containing 1.25 g IPTG, 1 g X-gal and 25 mL DMSO). The plaques were counted after an overnight incubation at 37 °C.

After 4 h incubation, the 20 mL *E. coli* ER2738 culture was centrifuged for 20 min at 2700 *g*, 4 °C. The supernatant was mixed with 7 mL PEG8000- 2.5 M NaCl to precipitate the phages and incubated overnight on ice. After a second incubation at 2700 *g*, 4 °C for 60 min, the phage containing pellet was redissolved in 1 mL TBS that were centrifuged for 5 min at 10,600 *g*. The supernatant was mixed with 200 µL PEG-NaCl and incubated on ice for 1 h to precipitate the phages. The precipitation was centrifuged for 45 min at 2600 *g*, the pellet was resuspended in 100 µL TBS (input). The input’s phage concentration was determined by spectrophotometry [41] in TBS using a 1:10 dilution. This procedure was repeated for all selection rounds. In selection round two and three, the respective input was also added to a well prepared in a similar fashion to the empty selection well and served as a direct control.

The single-stranded phage DNA was prepared for analysis by next generation sequencing (NGS) as described previously [42].

The data evaluation was performed with the software Target Sequencing Analysis Tool (TSAT) and Hammock [43], as described previously [42].

### 2.4. SPR Measurements

Binding studies were performed with surface plasmon resonance (SPR) in a Biacore T200 device (Biacore, GE Healthcare, Uppsala, Sweden). The L-ARQ46 was immobilized on a CM5 Chip via amine coupling (1400 RU). The selected D-enantiomeric peptide compound QF2D-2 was dissolved in the running buffer (TBS pH 7.5 with 0.05% Tween) and served as the analyte in multi-cycle experiments, with the highest peptide concentration being 20 µM. Experiments were performed at 25 °C with a flow rate of 45 µL/min. After a contact time of 110 s and a dissociation time of 400 s, the surface was regenerated with low pH (Glycin-HCl, pH 2.2) for 30 s with a flow rate of 30 µL/min. The data were evaluated via affinity fit of the steady state using the biacore evaluation software.

Additionally, K2Q46K2 was immobilized on a polycarboxylate-chip with 200 nm matrix (530 RU). The selected D-enantiomeric peptide compound QF2D-2 was dissolved in running buffer (TBS pH 7.5 with 0.05% Tween) and served as analyte in multi-cycle experiments. Experiments were performed at 25 °C with a flow rate of 20 µL/min. After a contact time of 140 s and a dissociation time of 400 s. The data were evaluated via affinity fit of the steady state using the biacore evaluation software.

### 2.5. Thioflavin T Assays

Thioflavin T (ThT) assays were performed to monitor the time-dependent formation of amyloidogenic aggregates. The assays were performed at 37 °C in TBS pH 7.5 (15 µM ThT) with 300 rpm double orbital shaking. All buffers were sterile filtrated beforehand. Polyglutamine proteins were disaggregated as described before. The peptides were pre-diluted in TBS pH 7.5. The disaggregated polyglutamine protein in buffer was mixed with 15 µM ThT and the pre-diluted peptides in a 96-well half-area flat-bottom microplate (Corning, New York, NY, USA). During the experiment all wells contained 100 µL ThT solution. Sealing the plate with foil (Thermo Fisher Scientific, Waltham, MA, USA) helped to prevent evaporation. The progression of fluorescence intensity was tracked by a microplate reader (BMG Labtech, Ortenberg, Germany).

The lag-time was estimated as follows: the average variation of the curve was determined with the last ten values of the steady state. Since the signal usually dropped at the beginning of the measurement, those values were not considered as starting point. As soon as the steady signal at the beginning exceeded the average variation, the lag-time was considered to be over.

For seeded experiments, the slope of the curve was considered instead of the lag-time because seeding eliminated the lag-time. The slope was determined by plotting the first values and fitting with a linear fit. The considered period (2 to 3.5 h) was similar for the curves compared with.

### 2.6. Production of Soluble Aggregate Fragments

The disaggregated ARQ46 was diluted to 300 µM and incubated at 37 °C, 400 rpm. After 24 h large, white aggregates were visible in the tube. The sample was sonicated (Sonopuls, Bandelin electronic GmbH & Co. KG, Berlin, Germany) four times for 15 s with an amplitude of 60%. Between the sonification steps the sample was cooled on ice. Post sonification the sample was turbid. It was centrifuged at 100,000 *g* for 1 h at 4 °C to remove insoluble aggregates from the solution. The clear supernatant was used for seeding with soluble aggregate fragments.

### 2.7. CD Spectroscopy

After disaggregation, polyglutamine proteins were dissolved in TBS pH 7.5 and measured in a quartz crystal cuvette with 1 mm light path (Hellma Analytics, Müllheim, Germany) in a CD spectrometer (J-1100, Jasco Deutschland GmbH, Pfungstadt, Germany) with 4 acquisitions. The High tension voltage (HT) was monitored throughout the experiment. Data for which the HT exceeded 600 V were excluded. Buffer measurements served as references. Analogous, samples containing 50 µM ARQ46 and 50 µM QF2D-1 or QF2D-2 were measured and referenced with the measurement of 50 µM QF2D-1 or QF2D-2 alone. Subsequently, the ARQ46 alone and with QF2D-1 or QF2D-2 were aggregated at 37 °C and 400 rpm for 7 days. The CD spectra of the samples were monitored after 24 h, 48 h and 168 h of aggregation.

## 3. Results

### 3.1. Aggregation and Disaggregation of Polyglutamine Proteins

In this study we followed a novel approach to identify therapeutics for protein misfolding diseases by stabilizing the protein’s native conformation with all-D-enantiomeric peptides as ligands. To this end it is essential to find binding partners of the monomeric target proteins.

**Figure 1:**
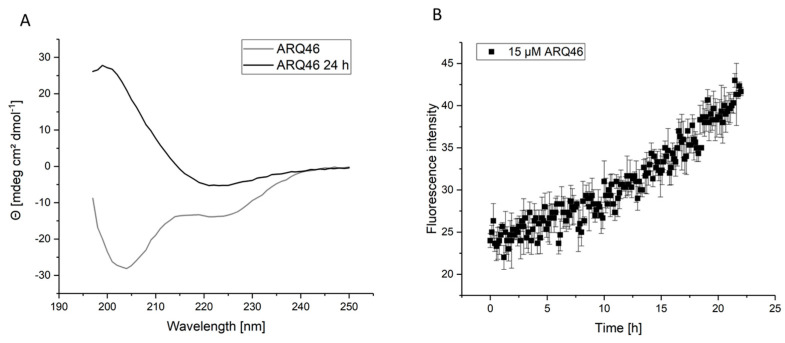
Disaggregation of ARQ46; Examination of ARQ46 post disaggregation. (**A**) CD-measurement of freshly disaggregated (100% TFA) ARQ46 (grey) in TBS pH 7.5 or the same sample incubated at 37 °C with 400 rpm shaking for 24 h (black). (**B**) ThT-Measurement of 15 µM ARQ46, disaggregated with 1:1 TFA/HFIP, in TBS pH 7.5 measured at 37 °C.

It is well known that polyglutamine proteins aggregate spontaneously when the number of glutamines exceeds a certain threshold [16]. This was observed for the tested constructs L-K2Q46K2 and L-ARQ46. Because of their spontaneous aggregation behavior, it was necessary to ensure that experiments were performed with monomeric polyglutamine proteins, whenever requested. The published disaggregation protocol [40] was adapted for the investigated polyglutamine proteins. Circular dichroism (CD) experiments with the disaggregated samples showed a mixture of random coil and alpha-helical structure (Figure 1A, grey curve). This is consistent with published CD-experiments [18]. After incubation at 37 °C, the CD measurement showed 100% beta-sheet structure (Figure 1A, black curve). It follows that the disaggregation protocol is successful and the protein was in its native conformation and capable of spontaneous misfolding after the disaggregation procedure.

This is supported by the Thioflavin T (ThT) aggregation assays, which showed that ARQ46 and K2Q46K2 (practically identical behavior, data not shown) spontaneously formed amyloid fibrils after few hours lag time, when incubated in TBS pH 7.5 at 37 °C (Figure 1B). The length of the lag time was dependent on the polyglutamine peptide’s concentration. It was observed that there was a longer and more reproducible lag-time in aggregation assays when ARQ46 was disaggregated in 1:1 TFA/HFIP. Lower concentrations of the ARQ46 then K2Q46K2 were required for similar aggregation results considering the lag time, which was eliminated by adding 5% seeds monomer equivalent.

### 3.2. Mirror Image Phage Display Selection

#### 3.2.1. Target Preparation

**Figure 2:**
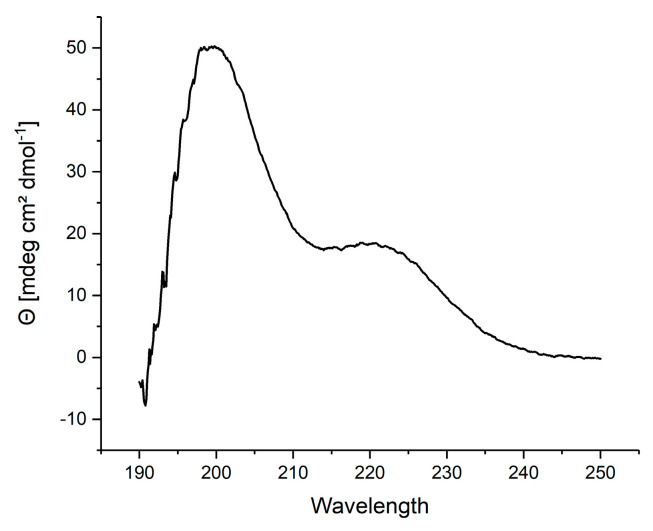
CD-spectrum of D-ARQ23 disaggregated with 100% TFA; Prior to the phage display selection D-ARQ23 was disaggregated with TFA to ensure selection on monomeric target. CD-spectrum was recorded in 10 mM Tris pH 8.

A mirror image phage display selection was performed to identify peptide ligands of polyglutamine proteins. To this end, a D-enantiomeric fragment of the androgen receptor comprised of amino acid residues 51 to 96 with a polyglutamine tract of 23 glutamines (D-ARQ23) was presented as target during the three selection rounds. An N-terminal biotin tag was connected via a ttds-linker to the D-ARQ23. Following the disaggregation protocol, with 100% TFA, the D-ARQ23 was disaggregated prior to the selection in order to assure its monomeric conformation. The disaggregation’s success was verified via CD spectroscopy. The CD sample was stored analogous to the target samples and measured after the selection’s completion to ensure the target stayed disaggregated for the selections time period. The resulting CD spectrum was opposite to the curve typical for disaggregated polyglutamine proteins (Figure 2), exactly as expected for a D-enantiomeric peptide.

#### 3.2.2. Selection and Evaluation

The selected phages from the phage library presenting 16mer peptides on their surface were analyzed via Next Generation Sequencing (NGS). The sequencing data were evaluated via TSAT, evaluation software developed by our work group [42] and the clustering tool Hammock [43]. There was no general sequence alignment of all selected sequences possible. The sequences were ranked by their empty score, which is the ratio between the frequency of the respective sequence in the target selection and the empty selection. To ensure comparability, the frequency was normalized to parts per million, because NGS runs might have different read numbers.

**Figure 3:**
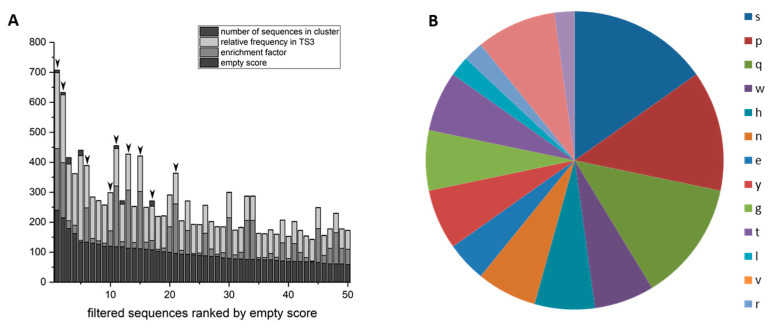
Sequences selected on D-ARQ23 ranked by empty score. (**A**) The sequences found by NGS sequencing were ranked by their empty score (deep dark grey). The sequences with the 50 highest empty scores are depicted. The enrichment factor (grey), relative frequency in target selection 3 (TS3; light grey) as well as the number of sequences with similar motives in the respective clusters (dark grey) are visualized within the bar chart. Sequences that were further investigated are marked with black arrows. (**B**) Amino acid residue type abundance in QF2D-1, QF2D-2 and QF2D-6.

In case the sequence is not present in the empty selection at all, half of the minimal empty value was used to calculate the empty score. The 50 sequences with the highest empty scores were compared regarding other parameters (Figure 3). One of those parameters is the enrichment factor, which is the ratio between the frequency in target selection round 3 and the frequency in the original library. If the sequence’s frequency in the library was zero, the half-minimal value was used, as described for the empty score. The ratio of target selection and direct control was also taken into account. It gives good hints concerning the phage’s target specificity, since the same input is presented to target or control well. If a sequence is more frequent in the target selection than the direct control it is highly probable that this sequence is a specific target binder. The last considered parameter was the formation of clusters, respectively sequences that are similar to each other. The Hammock tool was used to search the sequences for clusters. A cluster including many similar sequences can be used as a hint for target specific sequence enrichment. The selected sequences were also compared with those selected on a universal polyglutamine target, D-K2Q23K2. There were similarities in the preferred amino acid residues and also some sequences identical in both selections, another hint that this set-up is suitable to select specific polyglutamine binders.

Considering the described parameters, nine D-enantiomeric peptide sequences were chosen for further experimental investigations for their ability to inhibit the aggregation of polyglutamine proteins, marked with arrows in Figure 3. The peptides resulting from the marked sequences were named consecutively QF2D-1, QF2D-2 QF2D-3, QF2D-4, QF2D-5, QF2D-6, QF2D-7, QF2D-8 and QF2D-9. The sequences of the peptide and the considered parameters are shown in Table 2.

Overall, QF2D-1 had the highest empty score and enrichment factor. It was rare in the library and the leading sequence in a cluster with 8 other, similar sequences. It had a TS3/DC3 ratio bigger than 1, meaning it was more frequent in the target selection than in the direct control. The same was true for QF2D-2 that had the second highest empty score and a high enrichment factor. Similar to QF2D-1, it was rare in the library and found to be the leading sequence in a cluster. Compared to the other sequences it had the third highest ratio between Target selection and direct control.

The sequences with the third to fifth highest empty scores were excluded from further investigation, because they were also frequent in the library, meaning their enrichment factor was not as high.

We found that QF2D-3 had the sixth highest empty score and a high enrichment factor, as well as a high TS3/DC3 value, which is why it was tested.

The sequences with the seventh to ninth highest empty scores had low enrichment factors and were not included in any clusters; therefore they were not further investigated.

Additionally, QF2D-4 had the tenth highest empty score and was rare in the library, similar to QF2D-5, which was very rare in the library. QF2D-5 with the 11th highest empty score and had the second highest enrichment factor. It was also leading sequence in a cluster and had a high TS3/DC3 ratio. QF2D-7 was chosen for further investigation for the same reason.

However, QF2D-6 was also picked because of its high enrichment factor and the second highest TS3/DC3 ratio.

Moreover, QF2D-8 was the leading sequence in the cluster including the 18 sequences, whereas QF2D-9 had the highest TS3/DC3 ratio.

All other sequences were excluded, because their empty scores were not considered high enough.

By comparing the amino acid residue composition of the nine investigated peptides with the 50 leading sequences in the empty selection, it becomes apparent that three amino acid residues serine, proline and glutamine increased their proportion. Whereas serine, proline and glutamine make up 23% of the amino acid residues in the peptides selected in the empty selection, 33% of the amino acid residues in the nine selected peptides were serine, proline or glutamine. This becomes even more striking when we only consider the three peptides that had an effect in the seeded assay: QF2D-1, QF2D-2 and QF2D-6. 40% of the residues in these peptides are serine, proline or glutamine (Figure 3B). Their abundance nearly doubled in these peptides compared with the average of the empty selection.

Polyglutamine tracts modulate protein–protein interactions, especially between intrinsically disordered proteins [12]. It is not surprising that a glutamine rich sequence would be a preferred interaction partner for the monomeric polyglutamine target. Proline can interfere with the aggregation of polyglutamine proteins [44]. A proline-rich peptide could be effective in inhibiting the aggregation of polyglutamine proteins. The enrichment of specific residues compared to the empty selection is another hint that there was target-specific selection pressure that led to the enrichment of sequences with a high frequency of serine, proline and glutamine.

Three arginine residues were added C-terminally to the peptides that were further investigated to increase the peptide’s solubility. Arginines can increase cell membranes permeability [45].

### 3.3. Impact on Polyglutamine Aggregation

**Figure 4:**
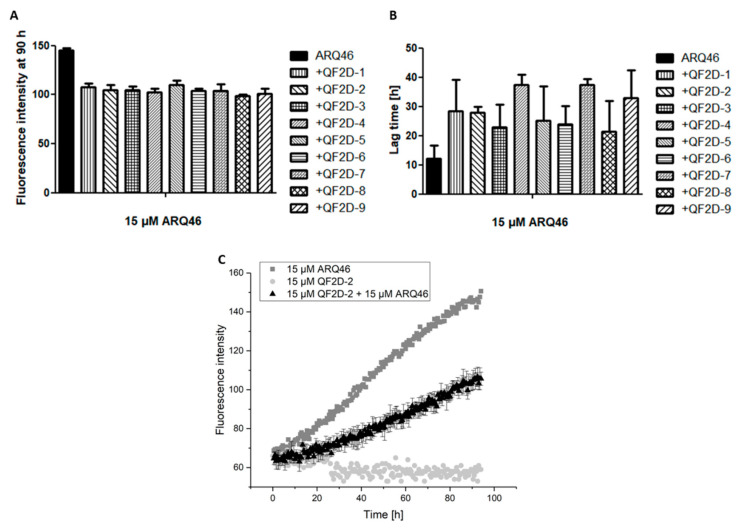
Compounds inhibit the aggregation of ARQ46; ThT assay with L-ARQ46 was performed at 37 °C in TBS pH 7.5. The progression of fluorescence intensity was measured every 30 min at λex = 410 nm and λem = 482 nm with 30 s agitation at 300 rpm before every measurement. (**A**) Fluorescence intensity measured at 90 h of the aggregation assay starting with 15 µM monomeric ARQ46 without peptide and with 15 µM QF2D-1, QF2D-2, QF2D-3, QF2D-4, QF2D-5, QF2D-6, QF2D-7, QF2D-8 or QF2D-9. (**B**) Lag time estimated from the aggregation assay of 15 µM monomeric ARQ46 without peptide and with 15 µM QF2D-1, QF2D-2, QF2D-3, QF2D-4, QF2D-5, QF2D-6, QF2D-7, QF2D-8 or QF2D-9. (**C**) Measurement example of the described ThT-assay with ARQ46 (dark grey) and QF2D-2 (black). QF2D-2 in TBS buffer alone served as a control (light grey). The mean fluorescence intensity is shown for each time point. The experiment was performed in three-fold determination.

In order to screen the selected all D-peptide compounds for their effect on polyglutamine aggregation, QF2D-1, QF2D-2, QF2D-3, QF2D-4, QF2D-5, QF2D-6, QF2D-7, QF2D-8 and QF2D-9 were tested via ThT-assay. The fluorescence intensity of ThT is proportional to amyloid content of the sample. In this first screening with equimolar ratios between peptides and ARQ46, all nine compounds had an inhibitory effect. All of them delayed ARQ46 aggregation and reduced the fluorescence intensity of the steady state (Figure 4). None of the compounds were ThT-active when no polyglutamine protein was present. For QF2D-9 this control was not evaluable, because the well dried during the experiment. Interestingly, all nine compounds reduced the fluorescence intensity of the steady state rather similarly to approx. 70% of the fluorescence intensity measured for ARQ46. There was higher variety among the compounds with respect to the lag-time elongation. The lag-time elongation varied between 76% (QF2D-8) and 207% (QF2D-7).

**Figure 5:**
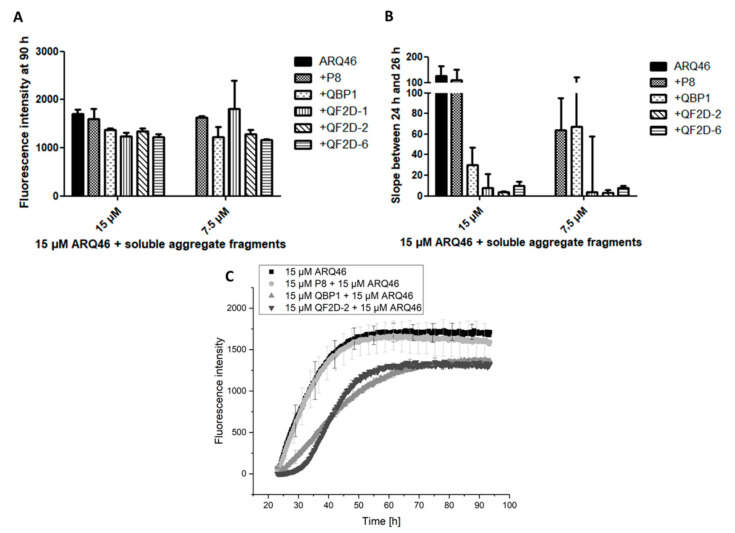
QF2D-1, QF2D-2 and QF2D-6 inhibit the aggregation of ARQ46 in presence of soluble aggregate fragment seeds; ARQ46 was aggregated, and the solution was subject to spinning at 100,000 *g* in an ultracentrifuge. The supernatant was preincubated with 15 µM and 7.5 µM of one of the selected compounds in TBS at 37 °C. After 23 h, 15 µM monomeric ARQ46 were added, and the fluorescence intensity was measured every 15 min at λex = 448 nm and λem = 482 nm with agitation at 300 rpm between measurements. P8 served as a control compound that was not selected on polyglutamine, whereas QBP1 is claimed to bind polyglutamine tracts and inhibit polyglutamine-driven aggregation. (**A**) Fluorescence intensity of P8, QBP1, QF2D-1, QF2D-2 and QF2D-6 in equimolar and sub stoichiometric ratio at 90 h. (**B**) The data points acquired between 24 h and 26 h- shortly after monomer addition- were fitted with a linear fit. The slope was compared. (**C**) Measurement example of the described ThT-assay with ARQ46 (black), P8 (light grey), QBP1 (grey) and QF2D-2 (dark grey). The mean fluorescence intensity is shown for each time point. The experiment was performed in two-fold determination.

Patients suffering from polyglutamine diseases present with aggregates in their neurons before symptom onset and therefore probably also before treatments start. Thus, a promising drug candidate should inhibit the aggregation in presence of pre-formed aggregates. Since small, soluble oligomers are suspected to be the main toxic species [46], the compounds were tested for their inhibitory effect in presence of soluble polyglutamine aggregate fragments (Figure 5). The effects of the compounds were compared with two controls. The first, P8, has been selected on SOD1 and should not bind to polyglutamine proteins. The second, QBP1, is claimed to inhibit the aggregation of polyglutamine proteins [33].

In presence of soluble aggregates, there was no lag-phase observable for the sample containing only ARQ46 or ARQ46 and P8. To compare the aggregation onset, the slope of the curve between 24 h and 26 h, so shortly after monomer addition, was calculated with a linear fit of the curves (Figure 5B). This slope represents the formation rate of amyloid aggregates by the increase of ThT-fluorescence per hour. The curves measured for ARQ46 and P8 were the steepest. QF2D-1, QF2D-2 and QF2D-6 had an aggregation inhibiting effect and decreased the slope, even when ARQ46 was present in excess. The effect of QF2D-1, QF2D-2 and QF2D-6 decreased the slope more effectively then QBP1, with QF2D-2 being the most and QF2D-6 being the least effective aggregation inhibiting compound. QF2D-2 reduced the slope by 96% compared with P8 and still by 86% compared to QBP1.

In the presence of soluble aggregate fragments, the effect of QBP1 was considerably smaller than that of the selected peptides. Since it is expected that a patient treated with a therapeutic agent for polyglutamine diseases already has aggregates formed in their neurons, the selected compounds might be more effective than QBP1 in this context.

**Figure 6:**
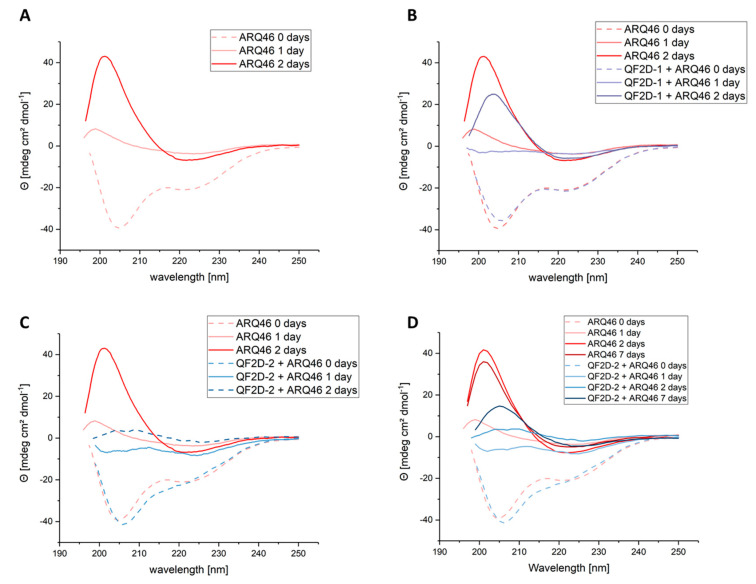
Investigation of structural change of ARQ46 in presence of QF2D-1 or QF2D-2 with CD spectroscopy; ARQ46 was disaggregated with 1:1 TFA/HFIP and diluted to 50 µM in TBS pH 7.5. 50 µM QF2D-1 or QF2D-2 were added to one sample. As a control and reference, 50 µM QF2D-1 or QF2D-2 alone were measured. (**A**) Spectra measured directly after disaggregation for 50 µM ARQ46 (rose, dashed)- referenced with buffer; 50 µM ARQ46 after 24 h incubation at 37 °C (rose, straight) and 50 µM ARQ46 after 48 h incubation at 37 °C (red, straight) (**B**) Spectra measured directly after disaggregation for 50 µM ARQ46 (rose, dashed)- referenced with buffer; equimolar ARQ46 and QF2D-1 (light purple, dashed)- referenced with QF2D-1 in TBS; 50 µM ARQ46 after 24 h incubation at 37 °C (rose, straight) and equimolar ARQ46 and QF2D-1 (light purple, straight)- referenced with QF2D-1 in TBS and 50 µM ARQ46 after 48 h incubation at 37 °C (red, straight) and equimolar ARQ46 and QF2D-1 (purple, straight)- referenced with QF2D-1 in TBS (**C**) Spectra measured directly after disaggregation for 50 µM ARQ46 (rose, dashed)- referenced with buffer and equimolar ARQ46 and QF2D-2 (light blue, dashed)- referenced with QF2D-2 in TBS; 50 µM ARQ46 after 24 h incubation at 37 °C (rose, straight) and equimolar ARQ46 and QF2D-2 (light blue, straight)- referenced with QF2D-2 in TBS and 50 µM ARQ46 after 48 h incubation at 37 °C (red, straight) and equimolar ARQ46 and QF2D-2 (light blue, straight)- referenced with QF2D-2 in TBS (**D**) Spectra measured directly after disaggregation for 50 µM ARQ46 (rose, dashed)- referenced with buffer; equimolar ARQ46 and QF2D-2 (light blue, dashed)- referenced with QF2D-2 in TBS; 50 µM ARQ46 after 24 h incubation at 37 °C (rose, straight) and equimolar ARQ46 and QF2D-2 (light blue, straight)- referenced with QF2D-2 in TBS; 50 µM ARQ46 after 48 h incubation at 37 °C (red) and equimolar ARQ46 and QF2D-2 (blue)- referenced with QF2D-2 in TBS; 50 µM ARQ46 after 168 h incubation at 37 °C (dark red) and equimolar ARQ46 and QF2D-2 (dark blue)- referenced with QF2D-2 in TBS.

During the aggregation process, the polyglutamine protein will change its secondary structure to a beta-sheet rich conformation. The selected compounds are expected to decelerate this transition by stabilizing the native conformation of the polyglutamine protein. The transition is monitored by CD spectroscopy. Freshly disaggregated, L-ARQ46 showed negative peaks at 222 nm and 205 nm, which is similar to the CD spectrum published by Chen et al. [18]. After 24 h incubation at 37 °C, the spectrum showed a positive peak at 200 nm range when no peptide was added to the sample. After 48 h incubation the spectrum showed that the sample was converted to beta-sheet [47,48] with a minimum at 220 nm and a maximum at 200 nm. (Figure 6A).

The sample containing QF2D-1 and ARQ46 (Figure 6B) did not show peaks in the positive or negative range after 24 h, probably because it was in the transition between the states since the CD spectrum is the median of the sample’s secondary structure. After two days of incubation, the sample with QF2D-1 showed a maximum at 205 nm, similar to ARQ46 alone, which had a maximum at 201 nm. This could be due to slightly different beta-sheet structures. It seems as if QF2D-1 decelerated ARQ46′s misfolding. The maximum at 205 nm is approx. 25% smaller than for ARQ46 alone, but after two days the transition seems to be complete as well.

This was not the case for the sample containing QF2D-2 and ARQ46 (Figure 6C,D). After 24 h incubation at 37 °C it still had a minimum at 205 nm although the minima’s amplitude was reduced compared to the spectrum of the freshly disaggregated sample. The transition to beta-sheet structures did not seem to have progressed as in the other samples at this time point. After 48 h at 37 °C this sample had a positive peak at 205 nm as well, but it was 90% smaller than that of ARQ46 alone, making it plausible that the sample is still partly in random coil conformation. Seven days after the disaggregation, the sample with QF2D-2 was refolded to beta-sheet as well. As observed for QF2D-1 the maximum was smaller than that of ARQ46 alone. Consistent with the aggregation study results, QF2D-2 seems to delay the transition to beta-sheet structures.

**Figure 7:**
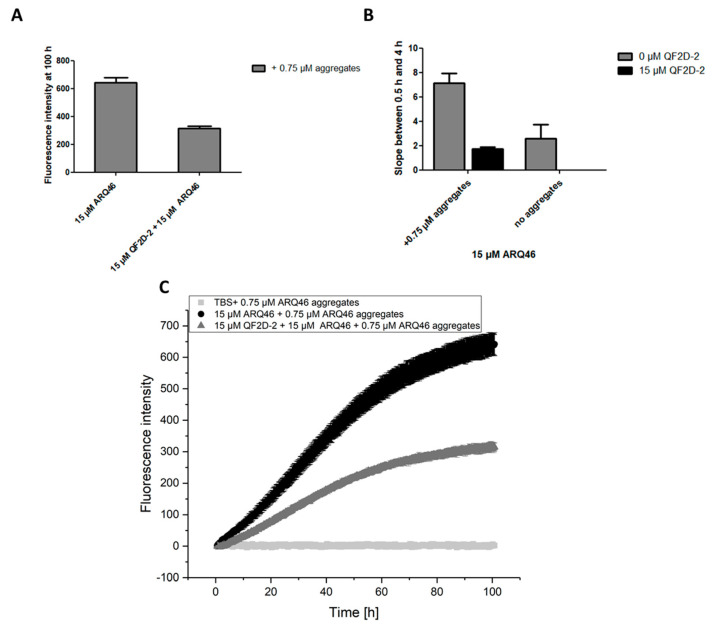
QF2D-2 inhibits the aggregation of ARQ46 in presence of soluble and insoluble seeds; ThT assay with L-ARQ46 was performed at 37 °C in TBS pH 7.5. Pre-formed ARQ46 aggregates were added in an amount of 5% monomer equivalent. The progression of fluorescence intensity was measured every 8 min at λex = 448 nm and λem = 482 nm with agitation at 300 rpm between measurements. (**A**) Fluorescence intensity measured at 100 h of the aggregation assay starting with 15 µM monomeric ARQ46, 0.75 µM soluble and insoluble ARQ46 aggregates and 15 µM QF2D-2. (**B**) The data points acquired between 0.5 h and 4 h were fitted with a linear fit. The slope was compared with the one measured for ARQ46 aggregation without seeds. (**C**) Measurement of ThT-fluorescence intensity with 15 µM ARQ46 (black) and 15 µM QF2D-2 (dark grey). 15 µM QF2D-2 with 0.75 µM ARQ46 aggregates served as control. The mean fluorescence intensity is shown for each time point. The experiment was performed in three-fold determination.

Summarizing the results so far, QF2D-2 seems to be the most promising candidate. Thus, its effect on ARQ46 aggregation was also tested when soluble and insoluble aggregates served as seeds. Here, the inhibiting effect of QF2D-2 was even stronger than the one on solely soluble aggregate seeded aggregation (Figure 7). The stronger effect might be due to large, insoluble fibrils that make up the majority of the seeds and partly precipitate out of the solution and therefore have a lower seeding capability than soluble aggregates [49]. Smaller aggregates might also have larger surfaces that can interact with the monomers. Furthermore, smaller fibril fragments will also present more ends for elongation than fewer, longer fibrils.

The aggregation experiment with monomeric ARQ46 was repeated with control compounds P8, which had a slight inhibiting effect on the aggregation when present in equimolar concentration, but the aggregation was similar to that of ARQ46 alone when P8 was present in a sub stoichiometric relation. When the aggregation experiment began with monomeric ARQ46, QBP1 drastically inhibited the aggregation, differing from the results of the seeded assay (Figure 5). QF2D-2 had an inhibiting effect, even when ARQ46 was in excess but in contrast to the seeded assay with soluble aggregate fragments the effect was not as strong as QBP1′s effect. QF2D-2 lowered the fluorescence intensity of the steady state and elongated the lag time compared to ARQ46 alone and with P8 (data not shown).

**Figure 8:**
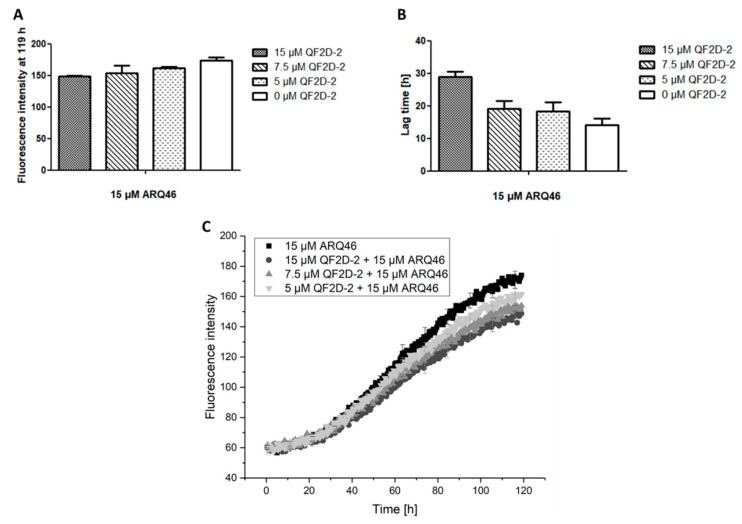
Effect of different QF2D-2 concentrations on the aggregation of ARQ46; ThT assay with L-ARQ46 was performed at 37 °C in TBS pH 7.5. The progression of fluorescence intensity was measured every 30 min at λex = 410 nm and λem = 482 nm with 30 sec agitation at 300 rpm before every measurement. (**A**) Fluorescence intensity measured at 119 h of the aggregation assay starting with 15 µM monomeric ARQ46 and 15 µM QF2D-2, 7.5 µM QF2D-2 or 5 µM QF2D-2. (**B**) Lag time estimated from the aggregation assay of 15 µM monomeric ARQ46 and 15 µM QF2D-2, 7.5 µM QF2D-2 or 5 µM QF2D-2. (**C**) Measurement example of the described ThT-assay with 15 µM ARQ46 (black), 15 µM QF2D-2 (dark grey), 7.5 µM (grey) and 5 µM QF2D-2 (light grey). The mean fluorescence intensity is shown for each time point. The experiment was performed in three-fold determination.

As described before, QF2D-2 inhibits aggregation when the polyglutamine protein is present in excess. This was verified in another aggregation experiment, showing that QF2D-2 had an inhibiting effect in a 1:3 ratio (QF2D-1:ARQ46). The effect was concentration dependend (Figure 8).

### 3.4. Binding to Polyglutamine Proteins

The binding properties of QF2D-2 to L-polyglutamine proteins were investigated by SPR. The QF2D-2 showed detectable binding to L-ARQ46. It bound with a K_D_ of 11 µM when L-ARQ46 was immobilized on the chip (data not shown).

This study aimed to identify ligands for polyglutamine-containing proteins. In order to investigate whether QF2D-2′s effect is constrained to the androgen receptor fragment used in the study, further SPR experiments were performed to investigate QF2D-2′s binding to more general polyglutamine constructs (L-K2Q23K2 and K2Q46K2). The glutamine flanking lysines were introduced to increase solubility in aqueous buffers [40].

**Figure 9:**
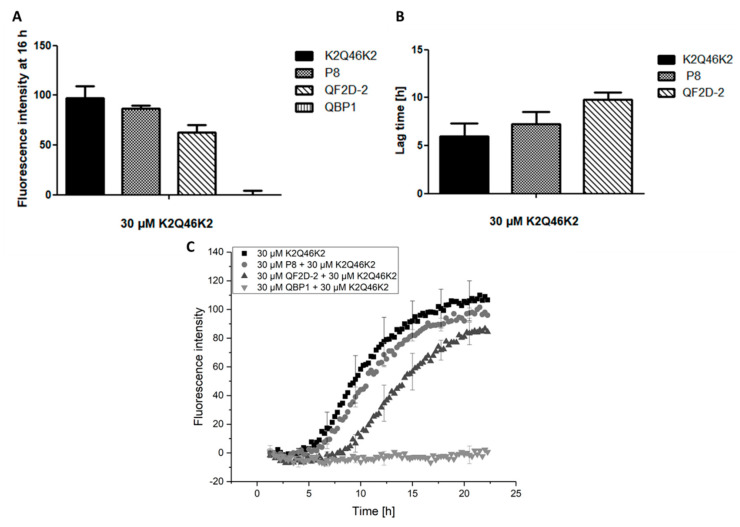
QF2D-2 inhibits the aggregation of K2Q46K2; ThT assay with L-K2Q46K2 was performed at 37 °C in TBS pH 7.5. The progression of fluorescence intensity was measured every 15 min at λex = 450 nm and λem = 480 nm with 300 s agitation at 300 rpm before every measurement. (**A**) Fluorescence intensity measured at 16 h of the aggregation assay starting with 30 µM monomeric K2Q46K2 and 30 µM QF2D-2, P8 or QBP1. (**B**) Lag times estimated from the aggregation assay of 30 µM monomeric K2Q46K2 and 30 µM QF2D-2 or P8. (**C**) Measurement example of described ThT-assay with K2Q46K2 (black) and QF2D-2 (dark grey) as well as P8 (grey) or QBP1 (light grey). The experiment was performed in three-fold determination.

Indeed, QF2D-2 bound to the general polyglutamine construct K2Q46K2 and inhibited its aggregation. It lowered the fluorescence intensity of the steady state and elongated the lag time compared to the effect of P8 (Figure 9). Thus, it may be feasible to transfer the compound’s inhibitory effect on aggregation to other polyglutamine containing proteins.

## 4. Discussion

The development of suitable therapeutics for SBMA is urgent, since no causative therapy is known. Instead of inhibiting or reducing the expression of the respective gene coding for the polyglutamine containing proteins [50,51,52], we aim to stabilize the protein’s native conformation by D-enantiomeric peptide ligands to prevent misfolding and aggregation, shift the equilibrium between aggregates and monomers towards monomers and dissolve the already existing aggregates into non-toxic and functional monomers. To this end a mirror image phage display selection was performed on a fragment of the androgen receptor, in which an elongated polyglutamine tract causes SBMA.

As is known, QBP1 is an L-enantiomeric peptide, which was selected on polyglutamine that had a therapeutic effect in *Drosophila* [34], but did not yield beneficial neurological effects in a respective mouse model [53,54,55].

L-enantiomeric peptide compounds metabolize rather quickly. Thus, we develop D-enantiomeric peptide ligands of polyglutamine proteins, a principle that was already shown to be effective in other neurodegenerative diseases [39]. D-enantiomeric peptides are, metabolically, considerably more stable [35], thus regular administration can lead to higher concentrations in the tissue.

## 5. Conclusions

An unphysiologically elongated polyglutamine tract causes protein misfolding and aggregation, leading to neurodegeneration. We performed a mirror image phage display selection on an all D-enantiomeric polyglutamine target to identify all D-enantiomeric peptide ligands for L-enantiomeric polyglutamine proteins. During the subsequent characterization of the all D-enantiomeric peptide compounds, we identified QF2D-2 to be the most promising among the nine compounds tested. Furthermore, QF2D-2 delayed the ThT-active aggregation of ARQ46, even under seeding conditions. CD spectroscopy measurements showed that QF2D-2 stabilizes ARQ46 in its native conformation and delays the transition to beta-sheet rich structures. Additionally, QF2D-2 not only bound to ARQ46 and inhibited its ThT-active aggregation, but also the more general target K2Q46K2, suggesting that it may be used and further developed as a more general compound, targeting and stabilizing polyglutamine containing proteins.

## Figures and Tables

**Table 1:** Synthetic peptides used.

Name	Sequence	Synthesized by
D-ARQ23	GasllllqqqqqqqqqqqqqqqqqqqqqqqetsprqqqqqqGedGs	JPT
L-ARQ46	GASLLLLQQQQQQQQQQQQQQQQQQQQQQQQ QQQQQQQQQQQQQQQQETSPRQQQQQQGEDGS	Peptides & elephants
L-K2Q46K2	KKQQQQQQQQQQQQQQQQQQQQQQQ QQQQQQQQQQQQQQQQQQQQQQQKK	Peptides & elephants
QF2D-1	Gnprmteqhqsypphmrrr	Caslo
QF2D-2	sqsqwstpqGkwshwprrr	Caslo
QF2D-3	hnipqklGvwpwpeerrrr	Caslo
QF2D-4	rsfdenswqqflGpGerrr	Caslo
QF2D-5	Gyptypyntqsisswlrrr	Caslo
QF2D-6	sstlmaypnysmqGnerrr	Caslo
QF2D-7	hhwntawdpfhsvrrr	Caslo
QF2D-8	hqrdpswvlyGesrivrrr	Caslo
QF2D-9	eyeqhvkwpwinnqqhrrr	Caslo
QBP1	SNWKWWPGIFD	Peptides & elephants
P8	YDTPKHKDKTWPMM	Caslo

**Table 2:** D-peptides selected on ARQ23 that were further investigated with three additional, C-terminal arginine residues.

Peptide Name	Sequence	Empty Score	Enrichment Factor	Frequency in Library	Cluster Size	TS3/DC
QF2D-1	Gnprmteqhqsypphmrrr	240	205	1.2	9	2.7
QF2D-2	sqsqwstpqGkwshwprrr	215	184	1.2	8	9
QF2D-3	hnipqklGvwpwpeerrrr	134	114	1.2	1	6.1
QF2D-4	rsfdenswqqflGpGerrr	120	51	2.4	1	1.7
QF2D-5	Gyptypyntqsisswlrrr	118	202	0	10	5
QF2D-6	sstlmaypnysmqGnerrr	113	194	0	1	10.3
QF2D-7	hhwntawdpfhsvrrr	112	191	0	1	2
QF2D-8	hqrdpswvlyGesrivrrr	108	31	3.7	18	1.4
QF2D-9	eyeqhvkwpwinnqqhrrr	100	85	1.2	1	55.4
